# Behavioral and neurodevelopmental outcome of children after maternal allopurinol administration during suspected fetal hypoxia: 5-year follow up of the ALLO-trial

**DOI:** 10.1371/journal.pone.0201063

**Published:** 2018-08-23

**Authors:** Job Klumper, Joepe J. Kaandorp, Ewoud Schuit, Floris Groenendaal, Corine Koopman-Esseboom, Eduard J. H. Mulder, Frank Van Bel, Manon J. N. L. Benders, Ben W. J. Mol, Ruurd M. van Elburg, Arend F. Bos, Jan B. Derks, on behalf of the ALLO-trial study group

**Affiliations:** 1 Department of Perinatology, University Medical Center, Utrecht, The Netherlands; 2 Julius Center for Health Sciences and Primary Care, University Medical Center, Utrecht, The Netherlands; 3 Department of Perinatology, Academic Medical Center, Amsterdam, The Netherlands; 4 Department of Perinatology, University Medical Center, Groningen, The Netherlands; TNO, NETHERLANDS

## Abstract

**Objective:**

To evaluate the long-term neurodevelopmental and behavioral outcome of antenatal allopurinol treatment during suspected fetal hypoxia.

**Study design:**

We studied children born from women who participated in a randomized double-blind placebo controlled multicenter study (ALLO-trial). Labouring women in whom the fetus was suspected to have fetal hypoxia were randomly allocated to receive allopurinol or placebo. At 5 years of age, the children were assessed with 2 parent reported questionnaires, the Ages and Stages Questionnaire (ASQ) and the Child Behavior Checklist (CBCL). A child was marked abnormal for ASQ if it scored below 2 standard deviation under the normative mean of a reference population in at least one domain. For CBCL, a score above the cut-off value (95^th^ percentile for narrowband scale, 85^th^ percentile for broadband scale) in at least one scale was marked as abnormal.

**Results:**

We obtained data from 138 out of the original 222 mildly asphyxiated children included in the ALLO-trial (response rate 62%, allopurinol n = 73, placebo n = 65). At 5 years of age, the number of children that scored abnormal on the ASQ were 11 (15.1%) in the allopurinol group versus 11 (9.2%) in the placebo group (relative risk (RR) 1.64, 95% confidence interval (CI): 0.64 to 4.17, p = 0.30). On CBCL 21 children (30.4%) scored abnormal in de allopurinol group versus 12 children (20.0%) in the placebo group (RR 1.52, 95% CI: 0.82 to 2.83, p = 0.18).

**Conclusion:**

We found no proof that allopurinol administered to labouring women with suspected fetal hypoxia improved long-term developmental and behavioral outcome. These findings are limited due to the fact that the study was potentially underpowered.

**Trial registration:**

NCT00189007 Dutch Trial Register NTR1383

## Introduction

Perinatal asphyxia is one of the major problems in obstetrics, carrying an incidence of 4–9 per 1000 live born neonates [1,2]. It can lead to hypoxic-ischemic encephalopathy (HIE) of the newborn, which is strongly associated with neonatal death, cerebral palsy and cognitive disability [3].

Therapeutic hypothermia has been proven to be the only effective treatment of HIE in term and late term newborns [4]. Besides hypothermia, pharmacological agents are currently being studied for the prevention of HIE in asphyxia. During hypoxia, the brain is not only damaged by direct ischemia, but also via the production of free oxygen radicals after reoxygenation and reperfusion [5]. Allopurinol, a xanthine-oxidase inhibitor, inhibits conversion of hypoxanthine and uric acid, thereby limiting the production of free oxygen radicals. At high concentrations, allopurinol scavenges free radicals such as hydroxyl, chelates free iron, and inhibits lipid peroxidation and heat shock factor expression [6]. Animal studies using allopurinol during hypoxia have shown cardio-and neuroprotective effects [7–9]. Allopurinol has only been tested in small human studies [10–12]. In these three human trials, allopurinol was administered postnatally. A Cochrane review from 2012 assessed that the available data are not sufficient to determine the effect of allopurinol on HIE in newborns (Composite of death or severe neurodevelopmental disability (risk ratio 0.78; 95% CI 0.56 to 1.08; risk difference -0.14; 95% CI -0.31 to 0.04))[13].

It has been stated earlier that treatment has less to no effect if the interval to the initiation of treatment is too long or if the brain damage is too severe [14]. Therefore, it is conceivable that earlier treatment provides the opportunity to limit the amount of free radical formation and subsequent reperfusion injury.

We recently reported the results of the ALLO-trial, in which we evaluated the effect of allopurinol administered antenatal to the mother where the baby had suspected fetal hypoxia, to prevent or reduce cerebral damage [15]. Results showed a non-significant difference in cord blood levels of S100B, a tissue specific biomarker for brain damage, of 44.5 pg/ml (IQR 20.2–71.4) in the allopurinol group versus 54.9 pg/ml (IQR 26.8–94.7) in the placebo group (difference in median -7.69; 95% CI -24.9 to 9.52). A post-hoc analysis showed a potential neuroprotective effect of allopurinol on the proportion of infants with a cord S100B value above the 75th percentile in girls (RR 0.37, 95% CI 0.14 to 0.99) but not in boys (RR 1.4, 95% CI 0.84 to 2.3) (treatment-gender interaction (p = 0.049)) [15]. However, studies so far have only assessed the short-term outcomes of neuroprotective effects of allopurinol. Long-term effects of severe HIE include serious motor disease, mental retardation and seizure disorders [16], whereas the long-term effects of moderate to mild HIE have been studied less. Handel *et al*. [17] have reported that individuals suffering from moderate HIE, a more heterogeneous group, can develop behavioural problems later in life.

The long-term outcome of children exposed to antenatal allopurinol has not been studied earlier. This study aims to determine the effects of antenatal allopurinol administration during suspected fetal hypoxia on the developmental and behavioral outcome of children at 5 years of age.

## Methods

### Participants

The study population consisted of the children born from the mothers who participated in the ALLO-trial from October 2009 to December 2011 in 12 hospitals in the Netherlands. The study protocol of the ALLO-trial is described elsewhere [18]. In short, women were eligible when they were in labour and suspected of having fetal hypoxia. Fetal hypoxia was defined as an abnormal or non-reassuring fetal heart rate trace (according to The International Federation of Gynecology and Obstetrics criteria), significant ST-wave abnormalities (detected by the STAN-monitor) or abnormal fetal blood scalp sampling (pH <7.20).

Women were randomly assigned in a double-blind fashion to receive either a single intravenous dose of 500 mg allopurinol or an intravenous placebo immediately before delivery. The dose of allopurinol was based on a study in healthy pregnancies [19], and on our pilot study [20]. Allocation was performed by an attending nurse who administered the contents of study drugs, as soon as a consenting patient met the inclusion criteria. The nurse carrying out the study protocol was not directly involved in the care of the woman in labour to ensure no delay in delivering the child. Allopurinol and placebo had exactly the same appearance. Participants, care providers and those who assessed outcomes remained blinded for allocation during follow-up. The primary outcome was the level of S100B, an extensively studied biochemical marker for brain-tissue damage and subsequent neurodevelopmental disabilities [21, 22, 23], in cord blood. The concentrations of S100B were compared between the two groups, as well as neonatal outcomes.

### Recruitment

The follow-up study took place between October 2014 and December 2016. We used two parent-completed questionnaires to assess the neurodevelopmental and behavioral outcome at 5 years age: the Ages and Stages Questionnaire 60-month (2^nd^ edition) [24] and the Child Behavior Checklist 1.5–5 years [25]. An additional questionnaire about general health, hospital admittance, medication use and other background information was also used. The use of validated questionnaires to assess the long-term neurodevelopmental outcome was announced in the original study protocol [18].

Participants were first contacted by telephone to announce the follow-up study at children’s age of 59 months. Informed consent to approach these women for children’s follow-up was part of the informed consent from the original ALLO-trial. If the women still wanted to participate, the questionnaires were sent by post. Participants were asked to fill out the three forms (general questionnaire, ASQ and CBCL) within 2 months. Parents who failed to return the questionnaires within the given time frame, were given multiple reminder phone calls.

### Ages and Stages Questionnaire

The Ages and Stages Questionnaire (ASQ), second edition, is a valid screening tool designed to detect developmental delay in children [24]. It is the most commonly used parent-completed developmental screener worldwide [26]. The ASQ contains 5 subdomains: communication, gross motor skill, fine motor skill, problem solving and personal-social behavior. Each domain consists of 6 questions, with a maximum score of 60 points per domain. The ASQ (2^nd^ edition) has been validated against a professionally administered tool, the Bayley Scales of Infant Development [24]. Furthermore, the ASQ-60 months has been validated in a Dutch low-risk population [27]. If questionnaires were not completely answered, scores were adjusted according to the ASQ manual. A questionnaire was marked abnormal if the score was ≥ 2SD below the normative mean of a reference population (as presented by Hornman et al. [27]) in at least one developmental domain. The ASQ manual recommends all questionnaires to be completed within the recommended age-frame (57 through 66 months).

### Child Behavior Checklist

The Child Behavior Checklist (CBCL) 1.5–5 years consists of 100 items concerning behavioral and emotional problems in children, assessed by parental perception [25]. Parents rate the child's behavior on a 3-point scale (*not true*, *somewhat or sometimes true*, and *very true or often true*), for behavior the child shows now or has shown in the previous 2 months. The CBCL informs on seven narrow syndrome scales (emotionally reactive, anxious/depressed, somatic complaints, withdrawn, sleep problems, attention problems and aggressive behavior) and two broadband scales (internalizing and externalizing problems).

For each scale a standardized *t*-score is calculated. The borderline cut-off points are the 95th percentile in narrowband scales, and the 85th percentile in the broadband scales. A score above the borderline cut-off point in either a broadband scale (*t*-score ≥ 60) or narrowband scale (*t*-score ≥ 65) was defined as abnormal and indicates behavioral problems.

#### Statistical analyses

The analysis was performed according to the intention-to-treat principle.

Baseline characteristics were compared using t-tests for continuous data, chi-square tests or Fisher’s exact tests (where appropriate) for dichotomous data and Mann-Whitney U-tests for non-normal distributed data. Mean scores of the CBCL and ASQ were compared using *t*-tests, and dichotomous data comparing the number of abnormal questionnaires per treatment group using chi-square tests. In addition, we conducted a post hoc subgroup analysis based on gender.

A log-binomial regression model was used to test for interaction of gender (p_interaction_) and treatment allocation (p_effect_). We performed a stratified analysis for gender regardless of the interaction term. We performed a multiple log-binomial regression analysis to assess the effect of allopurinol on abnormal ASQ and CBCL outcomes, to which we added all baseline characteristics for which differences between the groups were observed as covariates. We also added the main outcome of the birth-cohort trial, S100B protein levels, since we previously identified a small reduction in S100B for girls as compared to boys.

Data were analyzed using SPSS (version 21.0, IBM/SPSS Statistic, Armonk, NY, USA).

### Ethical approval

This follow-up study was approved by the local ethical committee of the University Medical Center Utrecht (The Netherlands) and the board of directors of the other participating centers as a part of the approval of the ALLO-trial. Parents of all participating children provided written informed consent.

## Results

**Fig 1:**
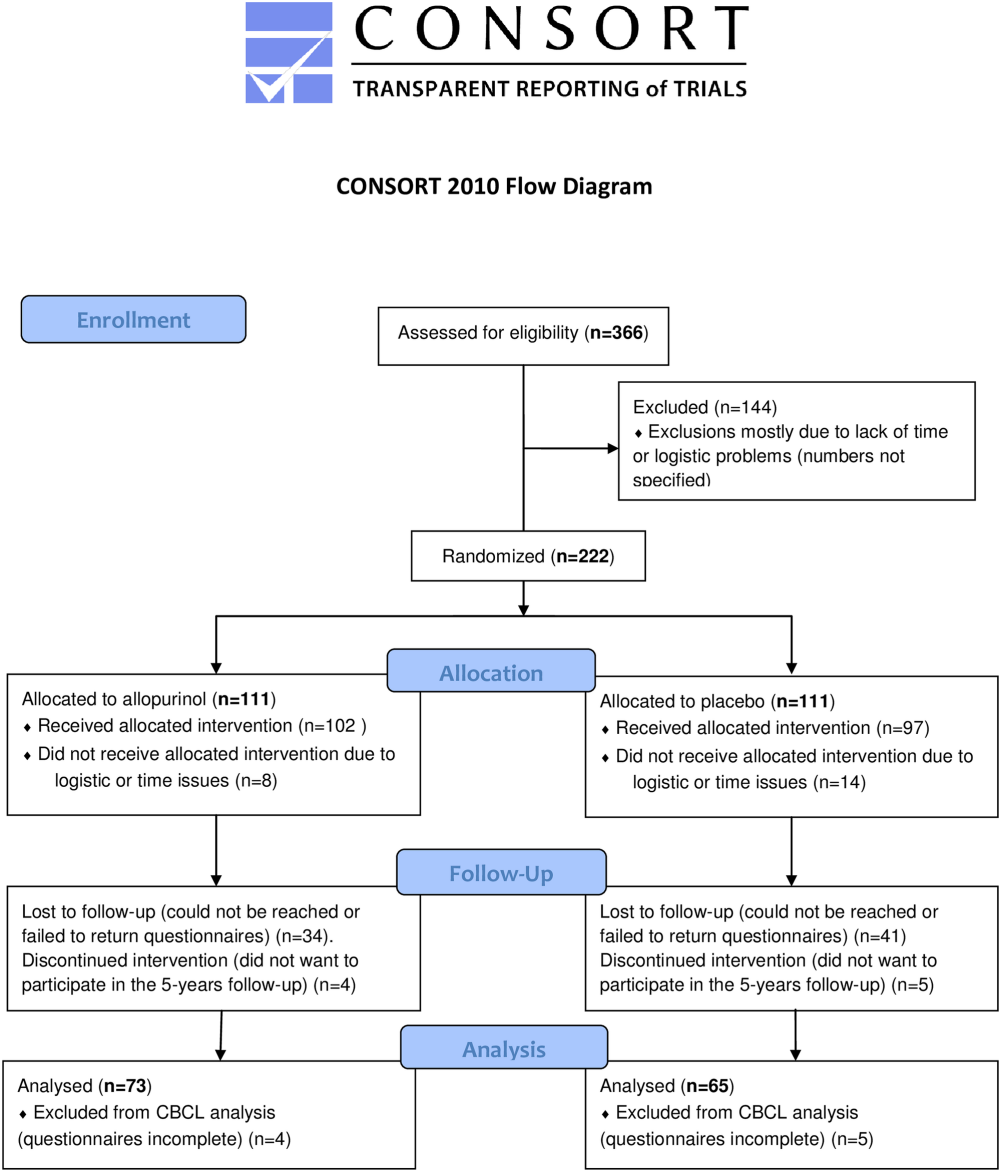
CONSORT flow diagram of the ALLO-trial and its follow-up. ALLO-trial: Antenatal allopurinol trial for reduction of birth asphyxia induced brain damage. ASQ: Ages and Stages Questionnaire, CBCL: Child Behavior Checklist.

Fig 1 depicts the CONSORT Flow Diagram of the ALLO-trial and its follow-up. In the ALLO-trial, 222 women were randomized to receive either allopurinol (n = 111) or placebo (n = 111). Every participant was eligible for follow-up. A total number of 222 woman were contacted, and 132 (62% follow-up percentage) were included in the follow-up study.

45 participants could not be contacted due to logistical reasons (i.e. incorrect phone number, change of address). 9 participants did not want to participate in the follow-up due to a variety of reasons. Even though frequent reminder phone calls were placed, 30 participants failed to return their questionnaires. 9 CBCL’s had to be discarded because the questionnaires were incomplete. All questionnaires were completed within the recommended age-frame (57 through 66 months) according to the ASQ manual.

### Study population/baseline

Baseline characteristics of the respondents (n = 138) and the nonrespondents (n = 84) are displayed in Table 1. The respondents were more likely to be Caucasian (p = 0.03) and multiparous (p = 0.03). Baseline characteristics of women in the allopurinol group (n = 73) and placebo group (n = 65) that responded were comparable. The median age at time of completion of the questionnaires was 60 months (range 58 to 62 months) for both groups (p = 0.43). The levels of S100B were comparable between both groups.

**Table 1:** Baseline characteristics.

Characteristics	Respondents n = 138	Nonrespondents n = 84	Difference in percent or mean (95% CI)	Allopurinol n = 73	Placebo n = 65	Difference in percent or mean (95% CI)
Maternal age (y) mean (SD)	32.7 (4.35)	32.4 (4.73)	0.30 (-1.55 to 0.96)	32.7 (4.6)	32.8 (4.4)	- 0.1 (-1.7 to 1.4)
Caucasian, n (%)	126 (91.3)	65 (81.3)^a^	10.0 (0.3 to 19.0)	66 (90.4)	61 (93.8)	-3.4 (-12.4 to 5.5)
Education level mother						
Low, n (%)	51 (37.0)	16 (44.4)^b^	-7.4 (-10.6 to 25.6)	23 (31.5)	18 (27.7)	3.8 (-19.0 to 11.4)
High, n (%)	87 (63.0)	20 (55.6)^b^		50 (68.5)	47 (72.3)	-3.8
Smoking, n (%)	12 (9.2)	8 (9.9)	-0.7 (-8.9 to 7.4)	8 (11.4)	4 (6.6)	4.9 (-4.8 to 14.6)
Nulliparity, n (%)	28 (20.3)	28 (33.3)	-13 (-25.2 to -0.9)	15 (20.5)	13 (20.0)	0.5 (-12.9 to 14.0)
Multiple gestation, n (%)	1 (0.7)	1 (1.2)	0.5 (-3.2 to 2.3)	1 (1.4)^c^	0 (0.0)	1.4 (-1.3 to 4.0)
Diabetes mellitus/gravidarum, n (%)	15 (10.9)	6 (7.1)	3.8 (-3.8 to 11.3)	7 (9.6)	8 (12.3)	-2.7 (-13.2 to 7.7)
PIH/PE, n (%)	37 (26.8)	18 (32.4)	-5.6 (-6.1 to 16.9)	21 (28.8)	16 (24.6)	4.2 (-10.6 to 18.9)
Gestational age at birth, median (IQR)	40.14 (38.82–41.14)	40.43 (39.18–41.14)	- 0.29^d^ (-0.3 to 0.9)	40.14 (38.5 to 41.1)	40.00 (38.9 to 41.2)	0.14^d^ (-0.8 to 1.1)
Infants^c^	138	84	NA	73	65	NA
Mode of delivery, n (%)						
Spontaneous	21 (15.2)	8 (9.6)	5.6 (-3.2 to 14.3)	15 (20.5)	6 (9.2)	11.3 (-0.3 to 23.0)
Instrumented vaginal delivery	63 (45.7)	38 (45.8)	0.1 (-13.7 to 13.4)	29 (39.7)	34 (52.3)	-12.6 (-29.1 to 4.0)
Caesarean section	54 (39.1)	37 (44.6)	-5.5 (-18.9 to 8.0)	29 (39.7)	25 (38.5)	1.3 (-15.0 to 17.6)
Gender						
Male, n (%)	90 (65.2%)	48 (57.1%)	8.1 (-5.2 to 21.3)	50 (68.5)	40 (61.5)	7.0 (-9.0 to 22.9)
Female, n (%)	48 (34.8%)	36 (42.9%)	-8.1	23 (31.5)	25 (38.5)	-7.0
S100B in umbilical cord (pg/mL), median (IQR)	46.4 (22.7 to 80.2)	48.1 (20.6 to 95.3)	-1.7^d^ (-13.3 to 10.0)	45.6 (24.3 to 78.5)	53.0 (21.7 to 81.3)	-7.4^d^ (-25.0 to 10.1)
Apgar score < 7 after 5 min, n (%)	9 (6.5)	5 (6.0)	0.5 (-6.0 to 7.1)	5 (6.8)	4 (6.2)	0.6 (-7.5 to 8.9)
pH umbilical cord < 7.05, n (%)^e^	5 (4.0)	1 (1.4)	2.6 (-1.7 to 7.0)	4 (5.9)	1 (1.8)	4.1 (-2.1 to 10.0)
Lactate in umbilical cord (mmol/L), mean (SD)^f^	6.85 (2.92)	6.86 (2.37)	0.01 (-0.82 to 0.83)	6.50 (2.55)	7.20 (3.23)	0.70 (-1.80 to 0.40)
NICU admission, n (%)	16 (11.6)	4 (4.8)	6.8 (-0.2 to 13.9)	10 (13.7)	6 (9.2)	4.5 (-6.1 to 15.0)
Birth weight (g), mean(SD)	3290 (563.0)	3243 (539.5)	47.0 (-198.1 to 104.2)	3240 (546.6)	3347 (579.8)	-107.8 (-297.4 to 81.9)
Treatment allocation						
Allopurinol, n (%)	73 (52.9)	38 (45.2)	7.7 (-5.9 to 21.2)	NA	NA	NA
Placebo, n (%)	65 (47.1)	46 (54.8)		NA	NA	NA

Table shows mean (SD) or number (percentile) unless specified otherwise. ‘Respondents’ group also includes incomplete questionnaires. ‘Nonrespondents’ includes: not approached for follow-up, not willingly to participate and failed to return questionnaires, see also Fig 1). ‘Low educational level’ refers to special education, primary school, pre-vocational secondary education (<12 years), senior general secondary education, pre-university education, or secondary vocational education (13–16 years). ‘High educational level’ refers to higher professional education or university (17+ years). CI: confidence interval, SD: standard deviation, IQR: interquartile range, PIH: pregnancy-induced hypertension, PE: pre-eclampsia, NICU: neonatal intensive care unit

^a^ 1.8% missing data

^b^ Indicates a characteristic with >20% missing data

^c^ One dichoriotic twin in the allopurinol group, but only one child participated in follow-up

^d^ shows difference in median

^e^ 11.3% missing data

^f^ 19.4% missing data

### General health

None of the children had died. In the allopurinol group, two children had been diagnosed with autism, of which one attended special education. In the placebo group, two children attended a medical kindergarten.

### Ages and Stages Questionnaire

The mean ASQ scores per domain were not different between the allopurinol group and the placebo group (Table 2). Eleven children (15.1%) in the allopurinol group scored abnormal, versus 6 children (9.2%) in the placebo group (RR 1.63, 95% CI 0.64 to 4.17, p = 0.30), (Table 3). No significant differences were seen in the number of abnormal scores per domain (defined as a score ≤ 1SD from the normative mean (Appendix A in S1 File) or ≤ 2 SD from the normative mean (Appendix B in S1 File).

**Table 2:** Mean scores for ASQ and CBCL in each domain/scale, compared between 2 groups.

Variable	Both groups	Allopurinol	Placebo	Difference in mean scores (95% CI)	p-value
*ASQ*	ASQ (n = 138)	*ASQ (n = 73)*	*ASQ (n = 65)*		
Communication	53.2 (57.3)	52.4 (7.4)	54.0 (7.1)	-1.6 (-4.1 to 0.9)	0.20
Gross motor	55.0 (7.0)	55.3 (7.0)	54.7 (7.0)	0.5 (-1.8 to 3.0)	0.61
Fine motor	49.8 (11.3)	49.1 (12.1)	50.7 (10.4)	-1.6 (-5.4 to 2.2)	0.41
Problem solving	55.2 (7.5)	55.1 (7.2)	55.4 (7.9)	-0.3 (-2.9 to 2.2)	0.80
Personal social	57.2 (5.0)	57.0 (5.2)	57.4 (4.8)	-0.4 (-2.1 to 1.2)	0.59
ASQ total score	275.0 (28.1)	270 (29.7)	271.6 (26.3)	-1.6 (-11.0 to 8.2)	0.77
*CBCL*	*Both groups (n = 129)*	*CBCL (n = 69)*	*CBCL (n = 60)*		
Emotionally reactive	53.6 (5.4)	53.4 (5.4)	53.8 (5.5)	-0.4 (-2.3 to 1.4)	0.64
Anxious/depressed	51.3 (3.0)	51.4 (2.9)	51.3 (3.1)	0.1 (-1.0 to 1.1)	0.98
Somatic complaints	54.1 (6.1)	54.4 (6.2)	53.9 (6.1)	0.5 (-1.6 to 2.6)	0.66
Withdrawn	53.0 (4.7)	52.9 (5.0)	53.0 (4.5)	-0.1 (-1.8 to 1.5)	0.90
Sleep problems	51.7 (5.5)	51.1 (6.6)	52.3 (3.9)	-1.2 (-3.0 to 0.8)	0.26
Attention problems	53.5 (5.7)	53.9 (6.3)	52.9 (4.9)	1.0 (-1.0 to 3.0)	0.31
Aggressive behavior	52.2 (4.4)	52.2 (4.1)	52.3 (4.7)	-0.1 (-1.6 to1.5)	0.95
Internalizing	46.1 (10.2)	46.0 (10.4)	46.2 (10.0)	-0.2 (-3.7 to 3.4)	0.92
Externalizing	45.6 (9.5)	45.7 (9.3)	45.6 (9.8)	0.1 (-3.2 to 3.4)	0.95
CBCL total t-score	44.6 (9.6)	44.2 (9.6)	45.0 (9.6)	-0.8 (-4.1 to 2.6)	0.66

ASQ: Ages and Stages Questionnaires, CBCL: Child Behavior Checklists, CI: confidence interval, RR: relative risk

**Table 3:** Number of children with abnormal scores of Ages and Stages Questionnaires and Child Behavior Checklists in ≥ 1 areas.

Questionnaire	Allopurinol, n (%)^a^	Placebo, n (%)^b^	RR (95% CI)	p-value
ASQ	11 (15.1)	6 (9.2)	1.63 (0.64 to 4.17)	0.30
CBCL	21 (30.4)	12 (20)	1.52 (0.82 to 2.83)	0.18

ASQ: Ages and Stages Questionnaires, CBCL: Child Behavior Checklists, CI: confidence interval, RR: relative risk

^a^ ASQ n = 73, CBCL n = 69

^b^ ASQ n = 65, CBCL n = 60

### Child Behavior Checklist

For the CBCL, 21 children (30.4%) in the allopurinol group and 12 (20%) in the placebo group had an abnormal score (RR 1.52, 95% CI 0.82 to 2.83, p = 0.18) (Table 3). There was no difference in mean scores between the two groups (Table 2), nor in the number of children with an abnormal score per scale (Appendix C in S1 File).

### Subgroup analysis

The subgroup analyses showed a treatment-gender interaction for abnormal ASQ (p = 0.040), but not for abnormal CBCL (p = 0.824) (Table 4). In boys, 10 out of 50 (20.0%) had an abnormal ASQ in the allopurinol group compared to 2 out of 40 (5.0%) in the control group (RR 4.0, 95% CI 0.93 to 17.2, p = 0.06). In girls, 1 out of 23 (4.3%) had an abnormal ASQ in the allopurinol group compared to 4 out of 25 (16.0%) in the placebo group (RR 0.27, 95% CI 0.03 to 2.26, p = 0.35.) In the CBCL, no significant differences for gender were found, see Table 4.

**Table 4:** Subgroup analysis. Number of children with abnormal scores of Ages and Stages Questionnaires and Child Behavior Checklists in ≥ 1 areas.

Questionnaire	Allopurinol, n (%)	Placebo, n (%)	RR (95% CI)	p-value	p-value for interaction
*ASQ*					
Boys (n = 90)	10 (20.0)	2 (5.0)	4.00 (0.93 to 17.2)	0.06	0.04
Girls (n = 48)	1 (4.3)	4 (16.0)	0.27 (0.03 to 2.26)	0.35	
*CBCL*					
Boys (= 85)	14 (29.8)	7 (18.4)	1.62 (0.73 to 3.60)	0.23	0.82
Girls (n = 44)	7 (32.8)	5 (22.7)	1.40 (0.52 to 3.74)	0.50	

ASQ: Ages and Stages Questionnaires, CBCL: Child Behavior Checklists, CI: confidence interval, RR: relative risk

We performed the regression analysis without additional covariates apart from S100B, since we did not identify any differences in baseline characteristics between the groups. The analysis did not show any association between allopurinol treatment or S100B protein levels and abnormal ASQ outcomes (p_effect_ = 0.58; p_interaction_ = 0.43) and CBCL outcomes (p_effect_ = 0.29; p_interaction_ = 0.95).

## Discussion

This follow-up study demonstrated that allopurinol administered to women in labour with suspected fetal hypoxia does not improve long-term developmental and behavioral outcome at 5 years of age.

Although results from previous research, both animal and human clinical studies, suggest a neuroprotective effect of allopurinol administered to neonates or fetuses during or after perinatal hypoxia [7–9,20,28], we were not able to confirm this in our follow-up study.

Emerging evidence suggests that gender differences play a role in the effectiveness of pharmacological neuroprotection after perinatal ischemia reperfusion [29]. Recent articles describe gender-dependent pathways of hypoxia-ischemia-induced cell death [30,31] or demonstrate the roll of sex hormones in neuroprotection [32,33]. The original ALLO-trial could only potentially suggest neuroprotection in girls, as indicated by the lower S100B and neuroketal levels in the treatment group. In this follow-up study, the subgroup analysis showed more abnormal scores in boys than in girls for behavioral outcome (ASQ), a non-significant difference. These results possibly illustrate the aforementioned gender-differences in a clinical setting. However, both studies were not designed to examine the differences in treatment effect, so the results should be interpreted with caution. In future studies concerning fetal neuroprotection during asphyxia, we suggest gender differences should be taken into account.

### Strengths and limitations

The ALLO-trial is a randomized, double blind placebo-controlled trial, and in this follow-up we maintained the blinding of treatment allocation of the parents and researchers.

Because of absent funding, only postal questionnaires were used to assess the outcome of the participating children. Although the ASQ and CBCL are parent-reported only, they have been validated as a solid screening tool and are widely used in obstetric follow-up studies.

A limitation to this study is its loss-to follow-up, with a response rate of 62.2%. Mothers who responded to the questionnaires were more frequently Caucasian and multiparous than nonrespondents. The number of women who smoked or had a low level of education (characteristics that are often associated with lower response-rate [34]) was the same in both groups. In the responders group, no significant differences in baseline characteristics were seen between the two study groups. Therefore, we feel that there is a low risk of selection bias.

Because the original study was a multicenter design, we could not to standardize the supportive care any child would receive during the follow-up period. One explanation of lacking differences between the two groups might be a recovery from developmental delay caused by perinatal asphyxia within 5 years by more intensive supportive care. Therefore, a difference in developmental outcome at 5 years of age could not be noticed. As reported in the ALLO-trial, the mean cord pH of the total group of infants, including the non-randomized patients, was 7.15, confirming the relatively mild hypoxia in the studied population, which is underlined by the mean lactate of 6.86 mmol/L. This lower than planned power for primary and secondary endpoints resulted in an unforeseen underpowered outcome of the ALLO-trial, and may have also led to an underpowered result of this follow-up study. Furthermore, it still is challenging to determine the severity of asphyxia antenatally. Perhaps a possible beneficial effect of allopurinol on reperfusion damage can be shown when allopurinol is administered to really hypoxic neonates directly after birth guided by umbilical cord arterial pH < 7.0 before neurological sequelae are visible.

Currently, a randomized placebo-controlled double blinded parallel group comparison study of hypothermia and allopurinol is ongoing in 13 different European countries, the ALBINO trial (EudraCT-number 2016-000222-19). Allopurinol, in addition to hypothermia, is administered to newborns with severe perinatal metabolic acidosis. The primary endpoint will be neurodevelopmental outcome 2 years age. It will facilitate further development and validation of biomarkers for neonatal brain injury using advanced magnetic resonance imaging, biochemistry, and electroencephalograms, which will then be available for future studies testing neuroprotective interventions.

## Conclusion

This 5-year follow-up study of the ALLO-trial showed no differences in neurodevelopmental and behavioral outcome of children exposed to antenatal allopurinol versus placebo in suspected fetal hypoxia. Future research will focus on postnatal neuroprotective interventions of allopurinol on more severely asphyxiated infants (ALBINO-trial).

## Supporting information

S1 File Appendix A, B, and C. (DOCX)

S2 File CONSORT checklist. (DOC)

S3 File Study protocol. (PDF)
