# Supplementary material for: Behavioral and neurodevelopmental outcome of children after maternal allopurinol administration during suspected fetal hypoxia: 5-year follow up of the ALLO-trial
Source: PLoS One. 2018 Aug 23;13(8):e0201063. doi: 10.1371/journal.pone.0201063 (PMC6107129; doi:10.1371/journal.pone.0201063)
Supplement: S1 File — (DOCX) [file pone.0201063.s001.docx]

**Appendix A: Number of abnormal ASQ scores per domain, defined as 1 SD under normative mean**

| **Domain** | **Allopurinol (n=73)** | **Placebo (n=65)** | **RR (95% CI)** | **p-value** |
| --- | --- | --- | --- | --- |
| Communication | 11 (15.1%) | 3 (4.6%) | 3.27 (0.95 to 11.19) | 0.051 |
| Gross motor | 11 (15.1%) | 7 (10.8%) | 1.40 (0.58 to 3.40) | 0.46 |
| Fine motor | 18 (24.7%) | 14 (21.5%) | 1.15 (0.62 to 2.11) | 0.67 |
| Problem solving | 8 (11.0%) | 7 (10.8%) | 1.02 (0.39 to 2.65) | 0.97 |
| Personal social | 5 (6.8%) | 3 (4.6%) | 1.48 (0.37 to 5.97) | 0.72 |

**Appendix B: Number of abnormal ASQ scores per domain, defined as 2 SD under normative mean**

| **Domain** | **Allopurinol (n=73)** | **Placebo (n=65)** | **RR (95% CI)** | **P value** |
| --- | --- | --- | --- | --- |
| Communication | 2 (2.7%) | 2 (3.1%) | 0.89 (0.13 to 6.14) | 1.00 |
| Gross motor | 3 (4.1%) | 2 (3.1%) | 1.34 (0.23 to 7.75) | 1.00 |
| Fine motor | 9 (12.3%) | 4 (6.2%) | 2.00 (0.65 to 6.20) | 0.25 |
| Problem solving | 3 (4.1%) | 1 (1.5%) | 2.67 (0.29 to 25.1) | 0.62 |
| Personal social | 3 (4.1%) | 2 (3.1%) | 1.34 (0.23 to 7.75) | 1.00 |

**Appendix C: Number of abnormal CBCL scores per scale. Cut-off according to CBCL manual**

| **Scale** | **Allopurinol (n=69)** | **Placebo (n=60)** | **RR (95% CI)** | **p-value** |
| --- | --- | --- | --- | --- |
| Emotionally reactive | 7 (10.1%) | 5 (8.3%) | 1.22 (0.41 to 3.64) | 0.72 |
| Anxious/depressed | 1 (1.4%) | 1 (1.7%) | 0.87 (0.06 to 13.60) | 1.00 |
| Somatic complaints | 10 (14.5%) | 6 (10.0%) | 1.45 (0.56 to 3.75) | 0.60 |
| Withdrawn | 2 (2.9%) | 1 (1.7%) | 1.76 (0.16 to 19.92) | 1.00 |
| Sleep problems | 1 (1.4%) | 0 (0.0%) | Not calculated | Not calculated |
| Attention problems | 7 (10.1%) | 3 (5.0%) | 2.03 (0.55 to 7.50) | 0.34 |
| Aggressive behavior | 1 (1.4%) | 2 (3.3%) | 0.44 (0.04 to 4.68) | 0.60 |
| Internalizing | 7 (10.1%) | 7 (11.7%) | 0.87 (0.32 to 2.34) | 0.78 |
| Externalizing | 7 (10.1%) | 5 (8.3%) | 1.22 (0.41 to 3.64) | 0.72 |

ASQ: Ages and Stages Questionnaires, CBCL: Child Behavior Checklists, RR: relative risk
