# Supplementary material for: Behavioral and neurodevelopmental outcome of children after maternal allopurinol administration during suspected fetal hypoxia: 5-year follow up of the ALLO-trial
Source: PLoS One. 2018 Aug 23;13(8):e0201063. doi: 10.1371/journal.pone.0201063 (PMC6107129; doi:10.1371/journal.pone.0201063)
Supplement: S3 File — (PDF) [file pone.0201063.s003.pdf]

Kaandorp et al. BMC Pregnancy and Childbirth 2010, 10:8
http://www.biomedcentral.com/1471-2393/10/8

BMC Pregnancy & Childbirth

STUDY PROTOCOL | Open Access

Antenatal allopurinol for reduction of birth asphyxia induced brain damage (ALLO-Trial); a randomized double blind placebo controlled multicenter study

Joepe J Kaandorp[1*], Manon JNL Benders[1], Carin MA Rademaker[2], Helen L Torrance[1], Martijn A Oudijk[3], Timo R de Haan[4], Kitty WM Bloemenkamp[5], Monique Rijken[6], Maria G van Pampus[7], Arie F Bos[8], Martina M Porath[9], Sidarto Bambang Oetomo[10], Christine Willekes[11], AW Danilo Gavilanes[12], Maurice GAJ Wouters[13], Ruurd M van Elburg[14], Anjoke JM Huisjes[15], Saskia CMJER Bakker[16], Claudia A van Meir[17], Jeannette von Lindern[18], Janine Boon[19], Inge P de Boer[20], Robbert JP Rijnders[21], Corrie JWFM Jacobs[22], Cuno SPM Uiterwaal[23], Ben Willem J Mol[3], Gerard HA Visser[1], Frank van Bel[1], Jan B Derks[1]

Abstract

Background: Hypoxic-ischaemic encephalopathy is associated with development of cerebral palsy and cognitive disability later in life and is therefore one of the fundamental problems in perinatal medicine. The xanthine-oxidase inhibitor allopurinol reduces the formation of free radicals, thereby limiting the amount of hypoxia-reperfusion damage. In case of suspected intra-uterine hypoxia, both animal and human studies suggest that maternal administration of allopurinol immediately prior to delivery reduces hypoxic-ischaemic encephalopathy.

Methods/Design: The proposed trial is a randomized double blind placebo controlled multicenter study in pregnant women at term in whom the foetus is suspected of intra-uterine hypoxia.
Allopurinol 500 mg IV or placebo will be administered antenatally to the pregnant woman when foetal hypoxia is suspected. Foetal distress is being diagnosed by the clinician as an abnormal or non-reassuring foetal heart rate trace, preferably accompanied by either significant ST-wave abnormalities (as detected by the STAN-monitor) or an abnormal foetal blood scalp sampling (pH < 7.20).
Primary outcome measures are the amount of S100B (a marker for brain tissue damage) and the severity of oxidative stress (measured by isoprostane, neuroprostane, non protein bound iron and hypoxanthine), both measured in umbilical cord blood. Secondary outcome measures are neonatal mortality, serious composite neonatal morbidity and long-term neurological outcome. Furthermore pharmacokinetics and pharmacodynamics will be investigated.
We expect an inclusion of 220 patients (110 per group) to be feasible in an inclusion period of two years. Given a suspected mean value of S100B of 1.05 ug/L (SD 0.37 ug/L) in the placebo group this trial has a power of 90% (alpha 0.05) to detect a mean value of S100B of 0.89 ug/L (SD 0.37 ug/L) in the 'allopurinol-treated' group (z-test$_{2\text{-sided}}$). Analysis will be by intention to treat and it allows for one interim analysis.

Discussion: In this trial we aim to answer the question whether antenatal allopurinol administration reduces hypoxic-ischaemic encephalopathy in neonates exposed to foetal hypoxia.

Trial registration number: Clinical Trials, protocol registration system: NCT00189007

* Correspondence: j.j.kaandorp@gmail.com
[1]Perinatal Center, University Medical Center, Utrecht, the Netherlands

BioMed Central

© 2010 Kaandorp et al; licensee BioMed Central Ltd. This is an Open Access article distributed under the terms of the Creative Commons Attribution License (http://creativecommons.org/licenses/by/2.0), which permits unrestricted use, distribution, and reproduction in any medium, provided the original work is properly cited.

Background

Hypoxic-ischaemic encephalopathy is associated with development of cerebral palsy and cognitive disability later in life [1]. The recognition, prevention and treatment of intra-uterine hypoxia is therefore one of the main priorities in perinatal medicine. Animal and human studies have shown that brain damage not only occurs during the hypoxic-ischaemic event, but continues for hours up to days upon and after reoxygenation and reperfusion and is caused by production of free radicals [2].

Free radical formation due to conversion of hypoxanthine into xanthine by xanthine-oxidase is very important during this process [3]. Administration of the xanthine-oxidase inhibitor allopurinol (ALLO) reduces the production of free radicals, thereby limiting the amount of hypoxia-reperfusion damage [4,5]. Furthermore, ALLO also has a non-protein bound iron (pro-radical) chelating and direct free radical (hydroxyl) scavenging effect. Animal research in asphyxiated pigs demonstrated beneficial effects of postnatally administrated ALLO on cerebral energy status and cytotoxic oedema [6].

A prospective randomized study in human neonates, examining the effects of ALLO in term asphyxiated neonates, showed an improvement of electrocortical brain activity and a reduction in free radical formation after neonatal ALLO administration [7]. A more recent paper by Gunes et al [8] reports an improved neurological outcome after postnatal ALLO administration compared to a placebo in term asphyxiated neonates. Benders et al however demonstrated that ALLO was not effective if administrated 3 to 4 hours after the hypoxic incident to severely asphyxiated neonates [9]. However, when the most severely asphyxiated children were excluded from the study, a beneficial effect of ALLO was seen on neurological development. This is in line with previous studies by Gluckman et al on neonatal head cooling [10]. They also demonstrated a beneficial effect of their treatment after exclusion of the most severely asphyxiated neonates. Apparently, no advantage of neonatal treatment is seen anymore, when the interval to the initiation of treatment is too long or when the brain damage is too severe. This has probably been the major disadvantage of late post neonatal treatment with ALLO on the Neonatal Intensive Care Unit (NICU). ALLO administrated at the NICU is likely to be given too late to provide adequate neuroprotection during the early period of reoxygenation in which the vast amount of free radicals is being produced.

Apparently, when the asphyxia has been too severe, the inflicted brain damage can no longer be reversed. It is conceivable that earlier ALLO treatment, i.e. the use of ALLO during labour in case of suspected foetal hypoxia, provides the opportunity to start earlier with the treatment, thereby limiting the amount of hypoxia-reperfusion injury and improving neurological outcome.

Pharmacological data have been published by Boda et al, who showed that pharmacological plasma levels were reached in the human foetus after ALLO administration to the mother [11]. A systematic review in Pubmed, searching for [allopurinol, foetus, neonate, asphyxia, hypoxia] did not provide us with any additional studies performed in humans. Some animal studies have been performed using piglets (neonatal administration), sheep and sows (maternal administration) [6,12-14]. These studies showed improved outcome after allopurinol treatment on cerebral energy status and recovery of umbilical blood flow respectively.

We recently performed a prospective randomized placebo controlled pilot study, in which we administered ALLO to the pregnant woman when foetal asphyxia was imminent. Data from this pilot study show an inverse correlation between levels of ALLO and the amount of S100B, a biomarker for brain tissue damage, in cord blood [15]. In addition, we performed a study in the chronically instrumented foetal sheep, in which we showed evidence of cardio- and neuroprotection after antenatal ALLO administration to the pregnant ewe during repeated periods of ischaemia [13,16].

Incidence and financial impact of birth asphyxia

Birth asphyxia carries a high incidence, 4-9 per 1000 live born neonates. On estimate 1-4 of these neonates will die or develop a severe handicap [17,18]. With almost 200.000 deliveries in the Netherlands annually, this implicates that each year approximately 800 neonates will die or suffer from a handicap due to birth asphyxia. Moreover, there is evidence that individuals suffering from moderate birth asphyxia, develop behavioural problems later in life [19].

The health care costs of asphyxia are enormous. Many of these children are admitted to a neonatal intensive care unit at a cost of € 1.500, - per day, with an average duration of admission of 10 days. Estimated costs of disabled children are € 80.000, - and € 20.000, - yearly for severely and moderately handicapped children respectively. The costs of neonatal care are annually 20 million Euros, whereas the costs of care for disabled children born in one year are more than one hundred million Euros.

As the costs of ALLO and its administration are relatively low (100 Euros per pregnant woman), a small treatment effect will already make the intervention cost-effective.

Safety of allopurinol

Allopurinol has been used in internal medicine for many years for the treatment of gout. Side effects of ALLO are

rare and most commonly include symptoms of hypersensitivity like skin rashes [20]. Stevens-Johnson syndrome has been reported, but only after prolonged treatment (> 10 days) in case of gout [21]. During previously performed studies in pregnant women and neonates, no adverse reactions were seen [11,15,22,23].

Methods/Design

Aims

The objective of the study is to test the hypothesis that intra-uterine treatment with ALLO in case of suspected foetal hypoxia will reduce brain damage and with that may improve neonatal outcome.

Participants/eligibility criteria

Pregnant women with a gestational age of at least 36 weeks and suspected intra-uterine hypoxia (foetal distress) during labour can be included in the trial. Foetal distress is being diagnosed by the clinician as an abnormal or non-reassuring foetal heart rate trace, preferably accompanied by either significant ST-wave abnormalities (as detected by the STAN-monitor) or an abnormal foetal blood scalp sampling (pH < 7.20). Neonates suspected of chromosomal or congenital anomalies will be excluded.

Procedures, recruitment, randomization and collection of baseline data

The study will be a randomized double blind placebo controlled multicenter study. Hospitals which participate in the Dutch Consortium for Studies in Women's health and reproductivity will participate in the trial. The study will be staffed by research midwives and -nurses, who will counsel patients at the outpatient clinic or at the obstetric ward.

Before entry into the study, subjects will be informed about the aims, methods, reasonably anticipated benefits and potential hazards of the study. Subjects will be informed that their participation is voluntary and that they may withdraw consent to participate at any time during the study. They will be informed that choosing not to participate will not affect their care. In every center an independent gynaecologist will be available for more detailed information both for patients and colleagues if required. After giving sufficient information, written informed consent will be obtained. The consent form must be signed before performance of any study-related activity.

In case of suspected foetal distress and written informed consent, the patient will be randomized immediately prior to delivery (Figure 1).

All details of delivery are recorded in the case record form that is accessible through a website http://www.studies-obsgyn.nl/allo. In case of admittance of one or more children to the neonatal intensive care unit, details of this admittance are also recorded.

Data will be collected using Oracle Clinical Remote Data Capture (RDC), which is a new generation of application systems that enable collection and cleanup of clinical trial data using the Internet. For detailed information on Oracle RDC see http://www.ctc-g.co.jp/~CTCLS/opa/en/. The expertise for this technology is already used in the study group and applied in the ZonMw funded consortium studies that are currently running.

Interventions

All women who are identified as having suspected foetal distress and have given informed consent prior to delivery will be randomized in two groups:

One group of pregnant women will receive one IV dosage of 500 mg ALLO. The other group will receive one IV dosage of placebo antenatally.

Outcome measures

Primary outcome measures are related to biochemical brain damage markers (S100B, enolase) and the severity of oxidative stress as measured by isoprostane, neuroprostane, non protein bound iron and hypoxanthine in umbilical cord blood.

The main primary outcome measure will be the protein S100B as this is, at present, the most validated biochemical marker for brain tissue damage.

Secondary outcome parameters are neonatal mortality and short term neonatal morbidity, such as hypoglycaemia, convulsions and post-hypoxic neonatal encephalopathy, length of admission at the neonatal intensive care unit and placental transfer, pharmacodynamics and -kinetics of ALLO. Neonatal encephalopathy will be assessed by Sarnat- and Thompson scores [24,25].

Follow up of women and infants

Blood sampling will be performed to assess the amount of tissue damage (i.e. S100B, enolase, troponin), oxidative stress (i.e hypoxanthine, isoprostane, neuroprostane and non protein bound iron) and ALLO plasma levels. Immediately after delivery, samples of cord blood and maternal blood are obtained. One hour and 18-24 hours after birth neonatal blood samples are obtained in all children with clinically indicated blood sampling.

Long-term follow-up using neuropsychological tests and validated neurodevelopmental questionnaires at the age of 4-5 and 8 years and additional MRI at the age of 8 years is desirable, but is depending on future funding.

Statistical issues

Sample size

The sample size is calculated based on the primary outcome measure cord-S100B as a marker of brain damage. Our own pilot study showed a mean value of S100B of

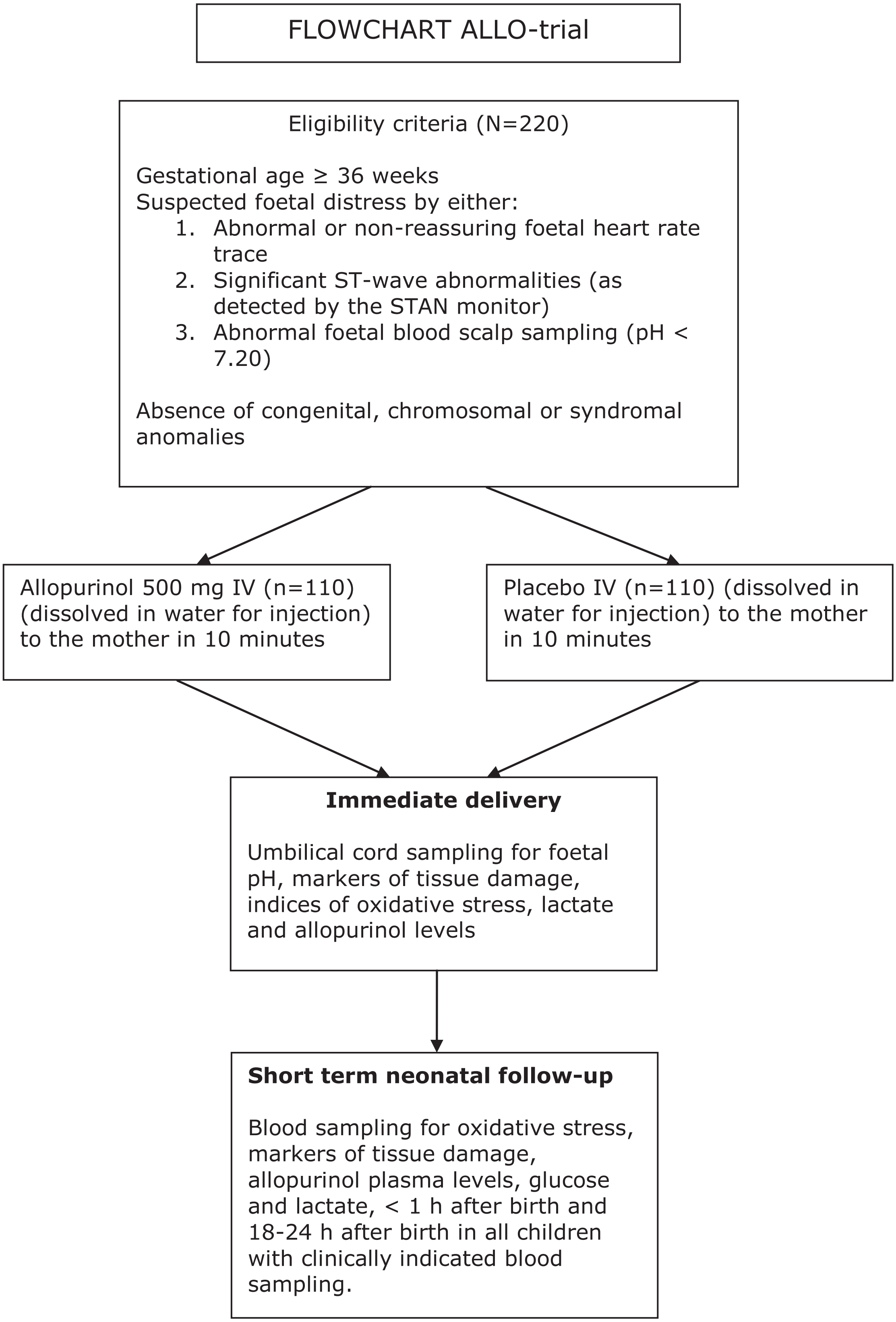

Figure 1 Flowchart ALLO-trial.

1.05 ug/L (SD 0.37 ug/L) in the 'non-treated' group with suspected intra-uterine hypoxia. Based on this same pilot study we expect the mean value of S100B to be lower in the 'allopurinol-treated' group compared to the 'non-treated group'.

Based on the accrual of patients in our pilot study we consider an inclusion of 220 patients (110 ALLO, 110 placebo) to be feasible in an inclusion period of 2 years. Given a suspected mean value of S100B of 1.05 ug/L (SD 0.37 ug/L) in the placebo group this trial has a power of 90% with an alpha of 0.05 to detect a mean value of S100B of 0.89 ug/L (SD 0.37 ug/L) in the 'allopurinol-treated' group (z-test$_{2\text{-sided}}$)[15].

Data analysis

Data will be analyzed according to the intention to treat principle. Outcome measures will be analyzed with (non)parametric tests where continuous variables are concerned. Proportional data will be reported as relative risks with 95% confidence intervals. Multivariable regression techniques will be applied to correct for any important differences in prognostic baseline characteristics, despite of randomization, by adding all prognostic variables as independent variables. Both corrected and uncorrected group differences will be reported. Numbers needed to treat (NNT) will be reported for both continuous and discrete data.

Interim analysis

An interim analysis will be performed at t = 0.5 using O'Brien -Fleming alpha spending function. An interim analysis will be performed after the inclusion of 110 women. This analysis will be done by an independent person that will be unaware of the allocation of treatment or placebo when they judge data on effectiveness.

Data safety monitoring committee

Serious Adverse Events (SAEs) and Suspected Unexpected Serious Adverse Reactions (SUSARs) will be reported to a Data Safety Monitoring Committee (DSMC). The DSMC can order to perform an extra interim analysis and, if indicated, terminate the trial prematurely.

Ethical considerations

This study is approved by the National Central Committee on Research involving Human Subjects (CCMO - NL26516.000.09).

Discussion

In conclusion, hypoxic-ischaemic encephalopathy is associated with development of cerebral palsy and cognitive disability later in life and is therefore one of the fundamental problems in perinatal medicine. Prevention of brain damage and developing adequate therapy is therefore of big importance. The xanthine-oxidase inhibitor allopurinol (ALLO) reduces free radical formation, thereby limiting the amount of hypoxia-reperfusion damage. Animal and human studies suggest that administration of ALLO immediately prior to delivery in case of suspected foetal asphyxia might reduce hypoxic-ischaemic encephalopathy. We designed a randomized placebo controlled multicenter trial to determine whether intra-uterine treatment with allopurinol reduces hypoxic-ischaemic encephalopathy in neonates exposed to foetal hypoxia.

To our knowledge no similar studies are or will shortly be performed in the Netherlands or abroad.

Abbreviations

ALLO: allopurinol; STAN: ST- waveform analysis; NICU: Neonatal Intensive Care Unit; SAEs: Serious Adverse Events; SUSARs: Suspected Unexpected Serious Adverse Reactions; DSMC: Data Safety Monitoring Committee

Acknowledgements

This study is funded by ZonMw, a Dutch organisation for Health Research and Development. Project number 170991001.

Author details

[1]Perinatal Center, University Medical Center, Utrecht, the Netherlands. [2]Department of Clinical Pharmacy, University Medical Center, Utrecht, the Netherlands. [3]Department of Obstetrics and Gynaecology, Academic Medical Center, Amsterdam, the Netherlands. [4]Department of Neonatology, Academic Medical Center, Amsterdam, the Netherlands. [5]Department of Obstetrics and Gynaecology, Leiden University Medical Center, Leiden, the Netherlands. [6]Department of Neonatology, Leiden University Medical Center, Leiden, the Netherlands. [7]Department of Obstetrics and Gynaecology, University Medical Center, Groningen, the Netherlands. [8]Department of Neonatology, University Medical Center, Groningen, the Netherlands. [9]Department of Obstetrics and Gynaecology, Máxima Medical Center, Veldhoven, the Netherlands. [10]Department of Neonatology, Máxima Medical Center, Veldhoven, the Netherlands. [11]Department of Obstetrics and Gynaecology, Maastricht University Medical Center, Maastricht, the Netherlands. [12]Department of Neonatology, Maastricht University Medical Center, Maastricht, the Netherlands. [13]Department of Obstetrics and Gynaecology, VU Medical Center, Amsterdam, the Netherlands. [14]Department of Neonatology, VU Medical Center, Amsterdam, the Netherlands. [15]Department of Obstetrics and Gynaecology, Gelre Hospital, Apeldoorn, the Netherlands. [16]Department of Paediatrics, Gelre Hospital, Apeldoorn, the Netherlands. [17]Department of Obstetrics and Gynaecology, Groene Hart Hospital, Gouda, the Netherlands. [18]Department of Paediatrics, Groene Hart Hospital, Gouda, the Netherlands. [19]Department of Obstetrics and Gynaecology, Diakonessenhuis, Utrecht, the Netherlands. [20]Department of Paediatrics, Diakonessenhuis, Utrecht, the Netherlands. [21]Department of Obstetrics and Gynaecology, Jeroen Bosch Hospital, 's Hertogenbosch, the Netherlands. [22]Department of Paediatrics, Jeroen Bosch Hospital, 's Hertogenbosch, the Netherlands. [23]Julius Center for Health Sciences and Primary Care, Utrecht, the Netherlands.

Authors' contributions

FvB, JD, MB, CR, HT, GV and BWM were involved in the conception and design of the study. JK, FvB, JD, MB, CR, CU and BWM drafted the manuscript. All authors mentioned in the manuscript are members of the ALLO-trial study group. They are local investigators at the participating centers. All authors read, edited and approved the final manuscript.

Competing interests

The authors declare that they have no competing interests.
There is no patent on allopurinol and therefore there is no interest by pharmaceutical companies to invest any funding in the proposed study. Both the allopurinol for IV administration and the placebo will be produced by a Dutch pharmaceutical company in Lelystad. Since there is no involvement or interest of the pharmaceutical industry, all results of the study will be available for publication.

Received: 23 October 2009
Accepted: 18 February 2010 Published: 18 February 2010

References

1. Fellman V, Raivio KO: **Reperfusion injury as the mechanism of brain damage after perinatal asphyxia.** *Pediatr Res* 1997, **41**:599-606.
2. Rosenberg AA, Murdaugh E, White CW: **The role of oxygen free radicals in postasphyxia cerebral hypoperfusion in newborn lambs.** *Pediatr Res* 1989, **26**:215-219.
3. Warner DS, Sheng H, Batinic-Haberle I: **Oxidants, antioxidants and the ischemic brain.** *J Exp Biol* 2004, **207**:3221-3231.
4. Palmer C, Vannucci RC, Towfighi J: **Reduction of perinatal hypoxic-ischemic brain damage with allopurinol.** *Pediatr Res* 1990, **27**:332-336.
5. Whitelaw A: **Systematic review of therapy after hypoxic-ischaemic brain injury in the perinatal period.** *Semin Neonatol* 2000, **5**:33-40.
6. Peeters-Scholte C, Braun K, Koster J, Kops N, Blomgren K, Buonocore G, van Buul-Offers S, Hagberg H, Nicolay K, van Bel F, Groenendaal F: **Effects of allopurinol and deferoxamine on reperfusion injury of the brain in newborn piglets after neonatal hypoxia-ischemia.** *Pediatr Res* 2003, **54**:516-522.
7. Van Bel F, Shadid M, Moison RM, Dorrepaal CA, Fontijn J, Monteiro L, van de Bor , Berger HM: **Effect of allopurinol on postasphyxial free radical formation, cerebral hemodynamics, and electrical brain activity.** *Pediatrics* 1998, **101**:185-193.
8. Gunes T, Ozturk MA, Koklu E, Kose K, Gunes I: **Effect of allopurinol supplementation on nitric oxide levels in asphyxiated newborns.** *Pediatr Neurol* 2007, **36**:17-24.
9. Benders MJ, Bos AF, Rademaker CM, Rijken M, Torrance HL, Groenendaal F, van Bel F: **Early postnatal allopurinol does not improve short term outcome after severe birth asphyxia.** *Arch Dis Child Fetal Neonatal Ed* 2006, **91**:163-165.
10. Gluckman PD, Wyatt JS, Azzopardi D, Ballard R, Edwards AD, Ferriero DM, Polin RA, Robertson CM, Thoresen M, Whitelaw A, Gunn AJ: **Selective head cooling with mild systemic hypothermia after neonatal encephalopathy: multicentre randomised trial.** *Lancet* 2005, **365**:663-670.
11. Boda D, Németh P, Kiss P, Orvos H: **Treatment of mothers with allopurinol to produce therapeutic blood levels in newborns.** *Prenat Neonat Med* 1999, **4**:130-134.
12. van Dijk AJ, Parvizi N, Taverne MA, Fink-Gremmels J: **Placental transfer and pharmacokinetics of allopurinol in late pregnant sows and their fetuses.** *J Vet Pharmacol Ther* 2008, **31**:489-495.
13. Derks JB, Oudijk MA, Rosén KG, Thakor AS, Visser GHA, van Bel F, Giussani DA: **Allopurinol maintains umbilical blood flow following repeated episodes of ischaemia-reperfusion in fetal sheep during late gestation [abstract].** *J Soc Gynecol Invest* 2006, **13**:s35.
14. Masaoka N, Nakajima Y, Hayakawa Y, Ohgame S, Hamano S, Nagaishi M, Yamamoto T: **Transplacental effects of allopurinol on suppression of oxygen free radical production in chronically instrumented fetal lamb brains during intermittent umbilical cord occlusion.** *J Matern Fetal Neonatal Med* 2005, **18**:1-7.
15. Torrance HL, Benders MJ, Derks JB, Rademaker CM, Bos AF, Berg Van Den P, Longini M, Buonocore G, Venegas M, Baquero H, Visser GH, Van Bel F: **Maternal allopurinol during fetal hypoxia lowers cord blood levels of the brain injury marker S-100B.** *Pediatrics* 2009, **124**:350-357.
16. Torrance H, Derks JB, Oudijk MA, Thakor AS, Cindrova-Davies T, van Bel F, Visser GHA, Burton GJ, Giussani DA: **Maternal treatment with allopurinol diminishes fetal cardiac oxidative stress following repeated episodes of ischaemia-reperfusion in sheep.** *Proceedings of the 34th annual meeting of the Fetal and Neonatal Physiological Society; 26-29 august 2007, Sendai, Japan* 2007, 90.
17. Ferriero DM: **Neonatal brain injury.** *N Engl J Med* 2004, **351**:1985-1995.
18. Hagberg B, Hagberg G, Olow I: **The changing panorama of cerebral palsy in Sweden. VI. Prevalence and origin during the birth year period 1983-1986.** *Acta Paediatr* 1993, **82**:387-393.
19. van Handel M, Swaab H, de Vries LS, Jongmans MJ: **Long-term cognitive and behavioral consequences of neonatal encephalopathy following perinatal asphyxia: a review.** *Eur J Pediatr* 2007, **166**:645-654.
20. **Summary of Product Characteristics Acepurin®.** http://db.cbg-meb.nl/Bijsluiters/h09974.pdf.
21. Halevy S, Ghislain PD, Mockenhaupt M, Fagot JP, Bouwes Bavinck JN, Sidoroff A, Naldi L, Dunant A, Viboud C, Roujeau JC, EuroSCAR Study Group: **Allopurinol is the most common cause of Stevens-Johnson syndrome and toxic epidermal necrolysis in Europe and Israel.** *J Am Acad Dermatol* 2008, **58**:25-32.
22. Chaudhari T, McGuire W: **Allopurinol for preventing mortality and morbidity in newborn infants with suspected hypoxic-ischaemic encephalopathy.** *Cochrane Database Syst Rev* 2008, **16(2)**:CD006817.
23. van Kesteren C, Benders MJ, Groenendaal F, van Bel F, Ververs FF, Rademaker CM: **Population pharmacokinetics of allopurinol in full-term neonates with perinatal asphyxia.** *Ther Drug Monit* 2006, **28**:339-344.
24. Sarnat HB, Sarnat MS: **Neonatal encephalopathy following fetal distress. A clinical and electroencephalographic study.** *Arch Neurol* 1976, **33**:696-705.
25. Thompson CM, Puterman AS, Linley LL, Hann FM, Elst van der CW, Molteno CD, Malan AF: **The value of a scoring system for hypoxic ischaemic encephalopathy in predicting neurodevelopmental outcome.** *Acta Paediatr* 1997, **86**:757-761.

Pre-publication history

The pre-publication history for this paper can be accessed here:http://www.biomedcentral.com/1471-2393/10/8/prepub

doi:10.1186/1471-2393-10-8
Cite this article as: Kaandorp *et al.*: Antenatal allopurinol for reduction of birth asphyxia induced brain damage (ALLO-Trial); a randomized double blind placebo controlled multicenter study. *BMC Pregnancy and Childbirth* 2010 **10**:8.

Submit your next manuscript to BioMed Central and take full advantage of:

- Convenient online submission
- Thorough peer review
- No space constraints or color figure charges
- Immediate publication on acceptance
- Inclusion in PubMed, CAS, Scopus and Google Scholar
- Research which is freely available for redistribution

Submit your manuscript at
www.biomedcentral.com/submit

BioMed Central
